# Supplementary material for: Iterative improvement in the automatic modular design of robot swarms
Source: PeerJ Comput Sci. 2020 Dec 7;6:e322. doi: 10.7717/peerj-cs.322 (PMC7924708; doi:10.7717/peerj-cs.322)
Supplement: Supplemental Information 3 [file peerj-cs-06-322-s003.zip › argos3/doc/api/standalone/a00532_source.html]

ARGoS: plugins/robots/generic/simulator/light\_default\_sensor.cpp Source File


- Main Page
- Related Pages
- Namespaces
- Classes
- Files

- File List
- File Members

# plugins/robots/generic/simulator/light\_default\_sensor.cpp

Go to the documentation of this file.

```
00001 
00007 #include <argos3/core/simulator/simulator.h>
00008 #include <argos3/core/simulator/entity/embodied_entity.h>
00009 #include <argos3/core/simulator/entity/composable_entity.h>
00010 #include <argos3/plugins/simulator/entities/light_entity.h>
00011 #include <argos3/plugins/simulator/entities/light_sensor_equipped_entity.h>
00012 
00013 #include "light_default_sensor.h"
00014 
00015 namespace argos {
00016 
00017    /****************************************/
00018    /****************************************/
00019 
00020    static CRange<Real> UNIT(0.0f, 1.0f);
00021 
00022    /****************************************/
00023    /****************************************/
00024 
00025    CLightDefaultSensor::CLightDefaultSensor() :
00026       m_bShowRays(false),
00027       m_pcRNG(NULL),
00028       m_bAddNoise(false),
00029       m_cSpace(CSimulator::GetInstance().GetSpace()) {}
00030 
00031    /****************************************/
00032    /****************************************/
00033 
00034    void CLightDefaultSensor::SetRobot(CComposableEntity& c_entity) {
00035       try {
00036          m_pcControllableEntity = &(c_entity.GetComponent<CControllableEntity>("controller"));
00037          m_pcLightEntity = &(c_entity.GetComponent<CLightSensorEquippedEntity>("light_sensors"));
00038          m_pcLightEntity->Enable();
00039       }
00040       catch(CARGoSException& ex) {
00041          THROW_ARGOSEXCEPTION_NESTED("Can't set robot for the light default sensor", ex);
00042       }
00043    }
00044 
00045    /****************************************/
00046    /****************************************/
00047 
00048    void CLightDefaultSensor::Init(TConfigurationNode& t_tree) {
00049       try {
00050          CCI_LightSensor::Init(t_tree);
00051          /* Show rays? */
00052          GetNodeAttributeOrDefault(t_tree, "show_rays", m_bShowRays, m_bShowRays);
00053          /* Parse noise level */
00054          Real fNoiseLevel = 0.0f;
00055          GetNodeAttributeOrDefault(t_tree, "noise_level", fNoiseLevel, fNoiseLevel);
00056          if(fNoiseLevel < 0.0f) {
00057             THROW_ARGOSEXCEPTION("Can't specify a negative value for the noise level of the light sensor");
00058          }
00059          else if(fNoiseLevel > 0.0f) {
00060             m_bAddNoise = true;
00061             m_cNoiseRange.Set(-fNoiseLevel, fNoiseLevel);
00062             m_pcRNG = CRandom::CreateRNG("argos");
00063          }
00064          m_tReadings.resize(m_pcLightEntity->GetNumSensors());
00065       }
00066       catch(CARGoSException& ex) {
00067          THROW_ARGOSEXCEPTION_NESTED("Initialization error in default light sensor", ex);
00068       }
00069    }
00070 
00071    /****************************************/
00072    /****************************************/
00073    
00074    void CLightDefaultSensor::Update() {
00075       /* Erase readings */
00076       for(size_t i = 0; i < m_tReadings.size(); ++i)  m_tReadings[i] = 0.0f;
00077       /* Ray used for scanning the environment for obstacles */
00078       CRay3 cScanningRay;
00079       CVector3 cRayStart;
00080       CVector3 cSensorToLight;
00081       /* Buffers to contain data about the intersection */
00082       SEmbodiedEntityIntersectionItem sIntersection;
00083       /* Get the map of light entities */
00084       CSpace::TMapPerTypePerId::iterator itLights = m_cSpace.GetEntityMapPerTypePerId().find("light");
00085       if (itLights != m_cSpace.GetEntityMapPerTypePerId().end()) {
00086          CSpace::TMapPerType& mapLights = itLights->second;
00087          /* Go through the sensors */
00088          for(UInt32 i = 0; i < m_tReadings.size(); ++i) {
00089             /* Set ray start */
00090             cRayStart = m_pcLightEntity->GetSensor(i).Position;
00091             cRayStart.Rotate(m_pcLightEntity->GetSensor(i).Anchor.Orientation);
00092             cRayStart += m_pcLightEntity->GetSensor(i).Anchor.Position;
00093             /* Go through all the light entities */
00094             for(CSpace::TMapPerType::iterator it = mapLights.begin();
00095                 it != mapLights.end();
00096                 ++it) {
00097                /* Get a reference to the light */
00098                CLightEntity& cLight = *any_cast<CLightEntity*>(it->second);
00099                /* Consider the light only if it has non zero intensity */
00100                if(cLight.GetIntensity() > 0.0f) {
00101                   /* Set ray end to light position */
00102                   cScanningRay.Set(cRayStart, cLight.GetPosition());
00103                   /* Check occlusions */
00104                   if(! GetClosestEmbodiedEntityIntersectedByRay(sIntersection,
00105                                                                 cScanningRay)) {
00106                      /* No occlusion, the light is visibile */
00107                      if(m_bShowRays) {
00108                         m_pcControllableEntity->AddCheckedRay(false, cScanningRay);
00109                      }
00110                      /* Calculate reading */
00111                      cScanningRay.ToVector(cSensorToLight);
00112                      m_tReadings[i] += CalculateReading(cSensorToLight.Length(),
00113                                                         cLight.GetIntensity());
00114                   }
00115                   else {
00116                      /* There is an occlusion, the light is not visible */
00117                      if(m_bShowRays) {
00118                         m_pcControllableEntity->AddIntersectionPoint(cScanningRay,
00119                                                                      sIntersection.TOnRay);
00120                         m_pcControllableEntity->AddCheckedRay(true, cScanningRay);
00121                      }
00122                   }
00123                }
00124             }
00125             /* Apply noise to the sensor */
00126             if(m_bAddNoise) {
00127                m_tReadings[i] += m_pcRNG->Uniform(m_cNoiseRange);
00128             }
00129             /* Trunc the reading between 0 and 1 */
00130             UNIT.TruncValue(m_tReadings[i]);
00131          }
00132       }
00133       else {
00134          /* There are no lights in the environment */
00135          if(m_bAddNoise) {
00136             /* Go through the sensors */
00137             for(UInt32 i = 0; i < m_tReadings.size(); ++i) {
00138                /* Apply noise to the sensor */
00139                m_tReadings[i] += m_pcRNG->Uniform(m_cNoiseRange);
00140                /* Trunc the reading between 0 and 1 */
00141                UNIT.TruncValue(m_tReadings[i]);
00142             }
00143          }
00144       }
00145    }
00146 
00147    /****************************************/
00148    /****************************************/
00149 
00150    void CLightDefaultSensor::Reset() {
00151       for(UInt32 i = 0; i < GetReadings().size(); ++i) {
00152          m_tReadings[i] = 0.0f;
00153       }
00154    }
00155 
00156    /****************************************/
00157    /****************************************/
00158 
00159    Real CLightDefaultSensor::CalculateReading(Real f_distance, Real f_intensity) {
00160       return (f_intensity * f_intensity) / (f_distance * f_distance);
00161    }
00162 
00163    /****************************************/
00164    /****************************************/
00165 
00166    REGISTER_SENSOR(CLightDefaultSensor,
00167                    "light", "default",
00168                    "Carlo Pinciroli [ilpincy@gmail.com]",
00169                    "1.0",
00170                    "A generic light sensor.",
00171                    "This sensor accesses a set of light sensors. The sensors all return a value\n"
00172                    "between 0 and 1, where 0 means nothing within range and 1 means the perceived\n"
00173                    "light saturates the sensor. Values between 0 and 1 depend on the distance of\n"
00174                    "the perceived light. Each reading R is calculated with R=(I/x)^2, where x is the\n"
00175                    "distance between a sensor and the light, and I is the reference intensity of the\n"
00176                    "perceived light. The reference intensity corresponds to the minimum distance at\n"
00177                    "which the light saturates a sensor. The reference intensity depends on the\n"
00178                    "individual light, and it is set with the \"intensity\" attribute of the light\n"
00179                    "entity. In case multiple lights are present in the environment, each sensor\n"
00180                    "reading is calculated as the sum of the individual readings due to each light.\n"
00181                    "In other words, light wave interference is not taken into account. In\n"
00182                    "controllers, you must include the ci_light_sensor.h header.\n\n"
00183                    "REQUIRED XML CONFIGURATION\n\n"
00184                    "  <controllers>\n"
00185                    "    ...\n"
00186                    "    <my_controller ...>\n"
00187                    "      ...\n"
00188                    "      <sensors>\n"
00189                    "        ...\n"
00190                    "        <light implementation=\"default\" />\n"
00191                    "        ...\n"
00192                    "      </sensors>\n"
00193                    "      ...\n"
00194                    "    </my_controller>\n"
00195                    "    ...\n"
00196                    "  </controllers>\n\n"
00197                    "OPTIONAL XML CONFIGURATION\n\n"
00198                    "It is possible to draw the rays shot by the light sensor in the OpenGL\n"
00199                    "visualization. This can be useful for sensor debugging but also to understand\n"
00200                    "what's wrong in your controller. In OpenGL, the rays are drawn in cyan when\n"
00201                    "they are not obstructed and in purple when they are. In case a ray is\n"
00202                    "obstructed, a black dot is drawn where the intersection occurred.\n"
00203                    "To turn this functionality on, add the attribute \"show_rays\" as in this\n"
00204                    "example:\n\n"
00205                    "  <controllers>\n"
00206                    "    ...\n"
00207                    "    <my_controller ...>\n"
00208                    "      ...\n"
00209                    "      <sensors>\n"
00210                    "        ...\n"
00211                    "        <light implementation=\"default\"\n"
00212                    "                   show_rays=\"true\" />\n"
00213                    "        ...\n"
00214                    "      </sensors>\n"
00215                    "      ...\n"
00216                    "    </my_controller>\n"
00217                    "    ...\n"
00218                    "  </controllers>\n\n"
00219                    "It is possible to add uniform noise to the sensors, thus matching the\n"
00220                    "characteristics of a real robot better. This can be done with the attribute\n"
00221                    "\"noise_level\", whose allowed range is in [-1,1] and is added to the calculated\n"
00222                    "reading. The final sensor reading is always normalized in the [0-1] range.\n\n"
00223                    "  <controllers>\n"
00224                    "    ...\n"
00225                    "    <my_controller ...>\n"
00226                    "      ...\n"
00227                    "      <sensors>\n"
00228                    "        ...\n"
00229                    "        <light implementation=\"default\"\n"
00230                    "                   noise_level=\"0.1\" />\n"
00231                    "        ...\n"
00232                    "      </sensors>\n"
00233                    "      ...\n"
00234                    "    </my_controller>\n"
00235                    "    ...\n"
00236                    "  </controllers>\n\n"
00237                    "OPTIONAL XML CONFIGURATION\n\n"
00238                    "None.\n",
00239                    "Usable"
00240                   );
00241 
00242 }
```

---

Generated on 10 Jul 2018 for ARGoS by 
 1.6.1
